# Synthesis of 2D Magnesium Oxide Nanosheets: A Potential Material for Phosphate Removal

**DOI:** 10.1002/gch2.201800056

**Published:** 2018-07-20

**Authors:** Saeed Ahmed, Arshad Iqbal

**Affiliations:** ^1^ State Key Laboratory of Chemical Resource Engineering Beijing Engineering Center for Hierarchical Catalysts Beijing University of Chemical Technology No. 15 Beisanhuan East Road, Chaoyang district Beijing 100029 China

**Keywords:** adsorption kinetics, magnesium oxide, nanosheets, phosphate removal, thermodynamics

## Abstract

Phosphate ions are responsible for eutrophication in drinking and wastewater, so it is necessary to limit the phosphate concentration in water bodies to limit the eutrophication problem. Porous magnesium oxide (MgO) nanosheets are synthesized at room temperature by simple precipitation and calcination. The synthesized material is characterized by various techniques. The sheet‐like MgO and commercial MgO are evaluated by the batch adsorption test. The synthesized material has an efficient adsorption efficiency of 95% at pH 5 as compared with commercial MgO, having a removal efficiency of 24% under the same investigated conditions. The synthesized MgO can be an efficient adsorbent material to overcome the eutrophication problem of the waste/domestic water.

## 1 Introduction

The eutrophication process by the algae growth has produced a lot of environmental problems. The decrease of phosphate in water bodies is an effective way to control the eutrophication process and reduce the algae growth in lakes and river.^1^ Phosphorus is an essential element in all the living systems, and it released in the form of phosphate from soils, rocks, industries, and household usage. Besides this, phosphate is also a necessary element for the growth of photosynthetic organism because it provides essential elements and energy,^2^, ^3^ but the excessive usage of phosphate resources and its discharge to the water bodies has created an alarming situation to the environment like blue algae growth in Taihu Lake in China.^4^

It is essential to limit the phosphate concentration in water bodies before discharge. In order to reduce the eutrophication process, different methods such as biological,^5^ chemical,^6^ and adsorption^7^ are used for the treatment purposes. Among these methods, adsorption is more effective, low‐cost technique for the phosphate removal. Typically, with the advancement of nanotechnology, various nanoadsorbent inorganic materials have been developed for the removal of phosphate.^8^, ^9^, ^10^ Among these materials, magnesium oxide materials have attained great attention in various applications of pollutant removal due to low cost and easy synthesis.^11^, ^12^ Magnesium oxide can be synthesized through various ways, with different properties depending upon the synthesis route,^13^, ^14^, ^15^ the structure, and pore properties which mainly depend upon the synthesis conditions.^16^, ^17^, ^18^

The purpose of this study is to utilize the magnesium salt to synthesize porous MgO at room temperature. Magnesium salts are widely available from the mineral sources extracted by the mining industry with high purity.^19^ The utilization of prepared porous MgO and commercial MgO is for the pollutant removal from aqueous solution.

## 2 Results and Discussion

### 2.1 Structure, Morphology, and Pore Properties

**Figure 1:**
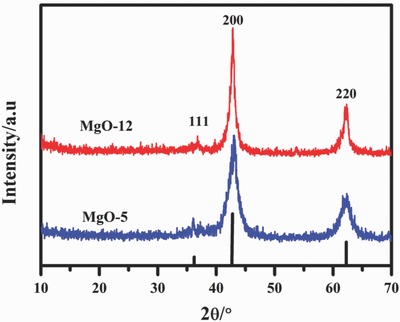
X‐ray diffraction pattern of MgO.

**Figure** **1** shows the X‐ray diffraction pattern of two prepared MgO samples. The three diffraction peaks at 36.86°, 42.94°, and 62.24°/2Ѳ in the range of 10–70°/2Ѳ correspond to the 111, 200, and 220, respectively. The geometry of MgO produced by this method represents the cubic closed packed structure. The crystallite size calculated by the well‐known Scherrer equation were 127 and 141 nm for the MgO‐5, and MgO‐12, respectively. The crystallite size increased with the passage of time due to increase in aggregation ratio. This shows that simple precipitation of Mg salt by ammonium carbonate is an available route for the low‐cost synthesis of MgO at room temperature.

**Figure 2:**
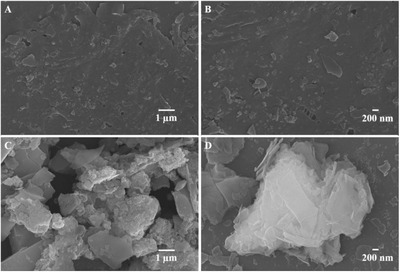
SEM images of A,B) MgO‐5 and C,D) MgO‐12.

**Figure** **2** shows the scanning electron microscope (SEM) images of two prepared MgO samples in which sheets of MgO were obtained with the thickness of about 50 nm which can be observed clearly in the SEM scans. The aggregation ratio of MgO sheets increased with the passage of time under constant stirring due to a decrease in reactants ratio in the reaction system. The 2D sheets generated building blocks of MgO, which can be clearly observed in the SEM scan of MgO‐12.

**Figure 3:**
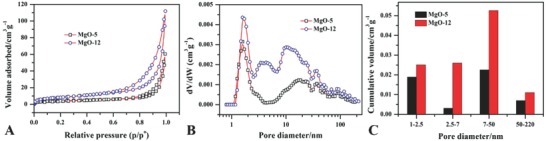
A) N_2_ adsorption desorption isotherm. B) Distribution of pore size. C) Cumulative pore volume distribution in different levels of pores for two MgO samples.

**Figure** **3**A shows the low‐temperature nitrogen desorption–adsorption isotherm for the two MgO samples. The isotherm is a typical III type with hysteresis loop of H4 which shows a sheet‐like structure with slit‐like pores. These results indicate that the aggregation of MgO sheets forms pores. These results are agreement with the SEM scan (Figure 2) for two MgO.^20^ Figure 3B shows that the two MgO samples exhibit different levels of pores including small mesopores (1–7 nm), large mesopores (7–50 nm), and macropores (50–225 nm). This pore size distribution in different levels for MgO is related to the slit combination by aggregation ratio to form pores of different levels and can be observed from SEM scan of two samples with the difference in aggregation ratio (Figure 3C).

**Table** **1** represents the pore parameters for the two MgO samples along with the adsorption capacity toward phosphate. The BET surface area varied from 15.32 for MgO‐5 to 30.13 m^2^ g^−1^ for MgO‐12, and pore size also varied from 24.28 to 22.97 nm for MgO‐5 and MgO‐12, respectively. A decrease in pore size is due to the blockage of some pores due to aggregate formation. From the above results, the BET surface area is high due to the more reaction time with aggregation ratio at room temperature for MgO‐12 as compared to hydrothermal treatment. These results show that the synthesis conditions played an important role in the formation and properties of MgO.

**Table 1:** Pore properties and adsorption capacity of phosphate for MgO

Sample	SBET [m^2^ g^−1^]	Pore volume [cm^3^ g^−1^]	Pore size [nm]	Adsorption capacity [mg g^−1^]
MgO‐5	15.32	0.09	24.38	165.29
MgO‐12	30.13	0.17	22.97	255.1

**Table 2:** Pseudo‐second‐order kinetic parameters for the three MgO samples at different thermodynamic temperatures

*T* [K]	MgO‐C			MgO‐5			MgO‐12		
	*q*_e,cal_ [mg g^−1^]	*K*_2_ [g mg^−1^ h^−1^]	*R* ^2^	*q*_e,cal_ [mg g^−1^]	*K*_2_ [g mg^−1^ h^−1^]	*R* ^2^	*q*_e,cal_ [mg g^−1^]	*K*_2_ [g mg^−1^ h^−1^]	*R* ^2^
298	37.37	0.06	0.99	102.15	0.02	0.99	196.46	0.001	0.97
303	44.98	0.04	0.99	136.80	0.01	0.99	245.1	0.004	0.99
308	61.69	0.03	0.99	165.29	0.01	0.99	255.1	0.01	0.99

### 2.2 Adsorption of Phosphate

#### 2.2.1 Effect of pH on the Removal Capacity

**Figure 4:**
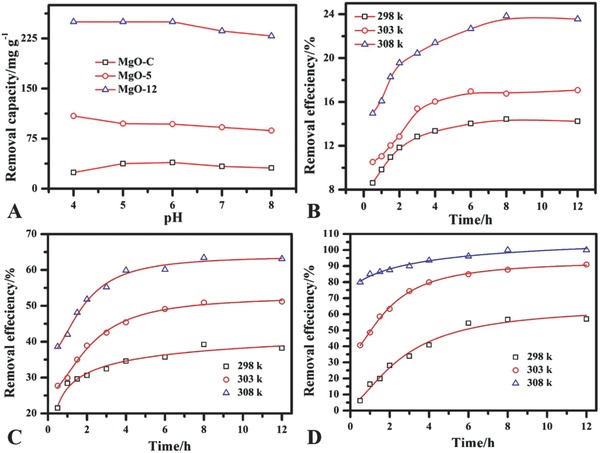
A) Effect of pH on the removal capacity of three MgO samples at 308 K. B) Effect of time on the removal efficiency of MgO‐C. C) Effect of time on the removal efficiency of MgO‐5. D) Effect of time on the removal efficiency of MgO‐12 at different thermodynamics temperatures when phosphate = 50 mg L^−1^, V = 100 mL, MgO = 20 mg, pH = 5.

Micro/nanostructures materials are very important for water treatment due to appropriate surface area and pore size which make them good adsorbent materials.^21^, ^22^, ^23^ In this work, we used commercial MgO and 2D MgO nanosheets for the adsorption of phosphate in an aqueous medium. The adsorption of cations and anions is generally affected by the initial pH of the experimental solution. In the present study, phosphate adsorption was also studied at different initial pHs. **Figure** **4**A shows the effect of pH (4–8) on the removal ability of MgO toward phosphate. The removal capacity decreased at a small level with an increase in solution pH for MgO‐C, and MgO‐5, while removal capacity remained constant in acidic range (4–6) for MgO‐12 and then gradually decreased at pH 7 and 8. MgO at acidic pH protonated to form more positive charge which can attract more phosphate anions toward the metal oxide materials.^24^, ^25^, ^26^

#### 2.2.2 Adsorption Kinetic

Figure 4B–D shows the removal efficiency of three MgO samples under the investigated conditions. The removal efficiency increased with the increase of contact time for all MgO samples. The equilibrium time for MgO‐C and MgO‐5 is about 8 h, while 6 h for the MgO‐12 with highest removal efficiency of 24%, 65%, and 95% for MgO‐C, MgO‐5, and MgO‐12, respectively, at 308 K.

**Figure 5:**
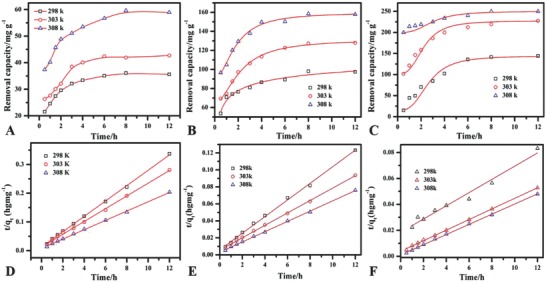
Effect of time on the removal efficiency at different thermodynamics temperatures for A) MgO‐C, B) MgO‐5, and C) MgO‐12 when phosphate = 50 mg L^−1^, V = 100 mL, MgO = 20 mg, and pH = 5. Second‐order kinetic of D) MgO‐C, E) MgO‐5, F) MgO‐12.

**Figure** **5**A,B shows the effect of time on the removal capacity of MgO‐C, MgO‐5, and MgO‐12 under the investigated conditions. The removal capacity of phosphate increased initially and slowed down with the passage of time due to occupancy of active sites. The removal capacity increased with the increase in thermodynamic temperature from 298 to 308 K for all MgO samples. This slow kinetic of phosphate adsorption is consistent with the studies reported which can be increased with the increase of adsorbent dosage.^27^ The calculated removal capacities for the MgO‐C, MgO‐5, and MgO‐12 are 61.69, 165.29, and 255.1 mg g^−1^ respectively (**Table** **2**). The pseudo‐second‐order kinetic model well fitted the adsorption of phosphate by commercial and the sheet‐like MgO. The correlation coefficients values for both samples at all temperatures are close to one (≥0.97). The second‐order model applicability shows the chemisorption process along with the physisorptions Table 2. The calculated removal capacities are also well consisted of the experimentally observed removal capacities. **Table** **3** represents the comparison of reported adsorbents materials with MgO toward phosphate.

**Table 3:** Comparison of different adsorbent materials toward phosphate adsorption

Adsorbent	pH	Pore size [nm]	*q*_max_ [mg g^−1^]	Ref.
ZrO2/SiO_2_	5	‐	43.8	35
Mg(OH)_2_	5	14.41	52.08	28
Hydrous MnO	7	‐	29.79	29
MgO	5	19.7	75.13	30
MgO	5	10.76	236	15
MgO	5	22.97	255.1	This work

#### 2.2.3 Effect of Temperature and Thermodynamics

**Figure 6:**
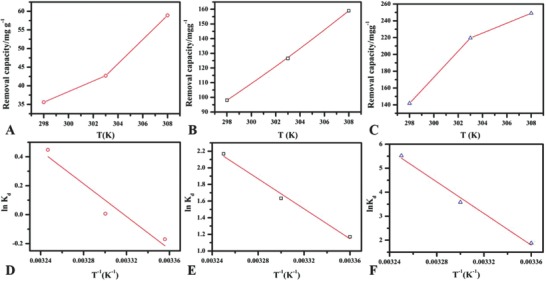
Effect of thermodynamic temperature on the removal capacity A) MgO‐C, B) MgO‐5 and C) MgO‐12 when phosphate = 50 mg L^−1^, pH = 5, V = 100 mL, equilibrium time = 8h, and linear plot of ln*K*_d_ versus 1/*T*, D) MgO‐C, E) MgO‐5, and F) MgO‐12.

**Figure** **6** shows the effect of thermostat temperature on the removal capacity toward phosphate under the investigated conditions; the experiments were performed at 298, 303, and 308 K for three MgO samples. Figure 6A shows the effect of temperature for MgO‐C, Figure 6B for MgO‐5, and Figure 6C for MgO‐12 in which removal capacity increased with the increase of thermostat temperature indicating the endothermic nature of MgO material toward phosphate adsorption which was similar to previously reported result for phosphate removal.^31^ This also shows that the phosphate adsorption is more favorable at high temperatures. The results of thermodynamic parameters are shown in **Table** **4**.

**Table 4:** Thermodynamic parameters of three MgO samples for phosphate adsorption

Sample	*T* [K]	*K*_d_ [mL g^−1^]	Δ*G* ^o^[KJ mol^−1^]	Δ*S*[KJ mol^−1^ K^−1^]	Δ*H*[KJ mol^−1^]
MgO‐C	298	0.84	420.64	156.01	47024.17
	303	1.01	−16.89		
	308	1.57	−1147.11		
MgO‐5	298	3.22	−2901.24	261.49	74985.89
	303	5.12	−4116.28		
	308	8.74	−5551.62		
MgO‐12	298	6.55	−4657.84	935.47	199147.71
	303	35.98	−9026.09		
	308	250	−14140.25		

Generally, the value of Gibb's free energy shows the spontaneity of the adsorption process. The value of the Gibb's free energy in this work at all temperatures is negative except for MgO‐C at 298 K, which shows that the phosphate adsorption is favorable and spontaneous while nonspontaneous at 298 K for MgO‐C. The positive Δ*H* value shows the endothermic nature of the phosphate adsorption, and positive value of the Δ*S* shows that the randomness of material increased with the increase in number of molecules during the adsorption process at an interface between MgO, and phosphate by the active sites.^31^, ^32^, ^33^

#### 2.2.4 Adsorption Mechanism

**Figure 7:**
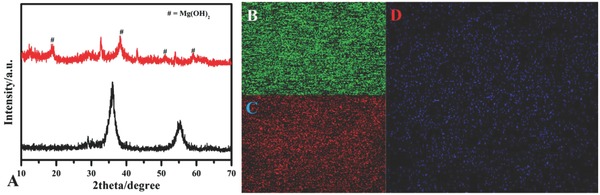
A) X‐ray diffraction before and after adsorption of phosphate for Mg‐12. Elemental mapping of MgO‐12 after adsorption of phosphate: B) magnesium, C) oxygen, D) phosphrus.

In order to understand the adsorption mechanism of phosphate, X‐ray diffraction and elemental mapping were done to confirm the adsorption process. **Figure** **7**A shows the X‐ray diffraction pattern of MgO‐12 sample before and after adsorption of phosphate. In comparison, the crystal structure of MgO has disappeared after adsorption, and new peaks of low intensity correspond to Mg(OH)_2_. Some other low intensity peaks in 12–38°/2Θ belong to Mg_3_(PO_4_)_2_.8H_2_O (JCPDS 33‐0877) and in 42–80°/2Θ belong to the Mg_3_(PO_4_)_2_.8H_2_O (JCPDS 33‐0329). These results show the chemical adsorption of phosphate along with the physical adsorption. Similar results were concluded from the kinetic data. Figure 7B–D shows the elemental mapping of the MgO‐12 after adsorption of phosphate belonging to magnesium and oxygen. Figure 6D belongs to phosphorus and shows that phosphate is uniformly distributed on the surface of MgO, and is also trapped into the MgO surface.^34^

## 3 Conclusion

In summary, we used a simple approach for the synthesis of porous 2D nanosheets of MgO by precipitation of magnesium nitrate using ammonium carbonate as a precipitating agent. This template‐free synthesis way provides low‐cost route to synthesize material exhibiting controlled morphology at room temperature. This highly porous material was proved to be efficient for the adsorption of phosphate as compared with the commercial MgO with removal efficiency of 95% along with the removal capacity of 255.1 mg g^−1^. Due to high efficiency of this material toward phosphate adsorption, MgO nanosheets are attractive adsorbent for water remediation to overcome the sustainable problems.

## 4 Experimental Section

*Synthesis of MgO*: Magnesium oxide was prepared by following two steps: 1) magnesium nitrate (0.2 mol L^−1^) and ammonium bicarbonate (1 mol L^−1^) were dissolved in 200 mL water separately through stirring. These solutions were mixed and continuously stirred in a beaker. After 5 min, half of the clear solution was transformed into a stainless steel autoclave and put in preheated oven at 120 °C for 6 h. After hydrothermal treatment, it was cooled down to room temperature and washed with deionized water several times, and dried at 80 °C for 12 h. This sample was marked as MgO‐5 in rest of the study. The remaining half solution was continuously stirred for 12 h, and white precipitates formed in solution were washed several times and this sample was marked as MgO‐12. These samples’ names were marked according to the stirring time while commercial MgO used in the adsorption of phosphate was marked as MgO‐C, 2) these dried samples were calcinated at 400 °C in air atmosphere for 4 h with a heating rate of 2 °C min^−1^.

*Batch Adsorption Experiment*: In order to investigate the effect of thermostat temperature on adsorption capacity towards phosphate anions on MgO surface, NaH_2_PO_4_ (one of the most present phosphate source salt in water bodies)^35^ was used for phosphate solution preparation. Stock solution (500 mg L^−1^) was prepared by dissolving the 0.2077 g of NaH_2_PO_4_.2H_2_O in 250 mL water.

In this study, solutions pH of 4–8 was studied by using conical flasks containing 100 mL of phosphate solution (50 mg L^−1^) that were placed in thermostat shaker for 12 h with shaking speed of 180 ± 5 rpm while pH was adjusted by the dropwise addition of 0.1 m NaOH/0.1 m HCl. Samples were collected at different time intervals and filtered by microfiltration membrane (0.45 µm) for the estimation of phosphate adsorption by molybdenum blue method.^36^ Removal capacity toward phosphate was calculated by using Equation (1) in which *V* is the volume of solution in L, *C*_o_ is the initial concentration while *C*_t_ is the concentration at any time *t*, and *m* is mass of MgO added.

(1) $$\mathrm{Removal}\;\mathrm{capacity}=\frac{V\left(C_{o}-\;C_{t}\right)}{m}$$

In order to investigate the effect of time on removal efficiency, 20 mg of each sample was dispersed in a 100 mL of a conical flask containing phosphate solution of 50 mg L^−1^. Phosphate solution pH was maintained at 5 ± 0.1 by adding a few drops of 0.1 m NaOH/0.1 m HCl. Conical flasks were placed in thermostat shaker at 298, 303, and 308 K with stirring speed of 180 ± 5 rpm. Removal efficiency was calculated by using Equation (2) in which *C*_o_ is the initial concentration while *C*_t_ is the concentration at any time *t*. Removal capacity at any time *t* was calculated by using Equation (1). The removal capacity data were fitted for pseudo‐second‐order rate Equation (3).

(2) $$\mathrm{Removal}\;\mathrm{efficiency}=\frac{V\left(C_{o\;}-\;C_{t}\right)}{\;C_{o}}\times 100$$

(3) $$\frac{C_{e}}{q_{e}}=\frac{1}{K_{2}q_{e}^{2}}+\frac{t}{q_{e}}$$

where *q*_e_ is the equilibrium removal capacity, *K*_2_ is the second‐order rate constant. These parameters values can be obtained from the linear plots.

The thermodynamics study was done at three different temperatures (298, 303, and 308 K) at the concentration of 50 mg L^−1^ at pH 5. Amount of phosphate adsorbed at equilibrium was determined by using Equation (1). Thermodynamic parameters can be calculated by using Equation (4)–(6), respectively.

(4) $$K_{d}=\;q_{e}/C_{e}$$

(5) $$\Delta G^{o}=\;-RT\;\ln\;K_{d}$$

(6) $$\ln K_{d}=-\Delta H/RT+\;\Delta S/R$$

where *K*_d_ (mL g^−1^) is the distribution coefficient and Δ*G* ^o^ (KJ mol^−1^) is Gibb's free energy. All the experiments were repeated twice; an average of two are presented in this work.

*Characterization*: Powder X‐ray diffraction patterns were recorded on (SHIMADZU XRD‐6000) X‐ray diffractometer using Cu Kα radiation operated at 40 KV from 3° to 70°/2theta with the scanning rate of 10°/2theta min^−1^. Zeiss Supra55 SEM was used to study the morphology of MgO. Low‐temperature N_2_ adsorption–desorption experiments were performed after a degassed treatment at 200 °C for 8 h on a Micrometrics (ASAP 2460) analyzer. Brunauer–Emmett–Teller (BET) method was used for the surface area while the pore volume and the pore size distribution were analyzed using the density functional theory method. The molybdenum blue method was used for quantitative determination of phosphate anions in aqueous solution by using UV–vis spectrophotometer Shimadzu (UV‐2501 PC).

## Conflict of Interest

The authors declare no conflict of interest.
